# Semi-Industrial Production of Kashkaval of Pindos Cheese Using Sheep or a Mixture of Sheep–Goat Milk and Utilization of the Whey for Manufacturing Urda Cheese

**DOI:** 10.3390/foods9060736

**Published:** 2020-06-03

**Authors:** Eleni C. Pappa, Efthymia Kondyli, Loulouda Bosnea, Marios Mataragas, Agathi Giannouli, Maria Tsiraki

**Affiliations:** Dairy Research Department, Hellenic Agricultural Organization-DEMETER, 45221 Katsikas, Ioannina, Greece; kondyliefi@gmail.com (E.K.); louloudabosnea@gmail.com (L.B.); mmatster@gmail.com (M.M.); agigiannouli@yahoo.gr (A.G.); tsir_maria@yahoo.gr (M.T.)

**Keywords:** traditional cheeses, semi-industrial manufacture, sheep, goat, milk, whey

## Abstract

Kashkaval of Pindos cheese was successfully produced using 100% sheep milk (KS) or with the addition of 10% goat milk (KG). Urda cheese was manufactured using 100% sheep (US) or 90% sheep–10% goat (UG) whey from the production of kashkaval of Pindos cheese. Both cheeses were made taking into account their traditional cheese-making methods. The cheeses were assessed for their chemical, microbiological and organoleptic characteristics. Generally, no significant differences were observed between KS and KG cheese and between US and UG cheese regarding their physicochemical, textural characteristics, soluble nitrogen fraction and total fatty acid content. The fat content of Urda cheese was low, in accordance with the demand of consumers for healthy products. KS cheeses showed higher total volatile compounds than KG cheeses at 60 and 90 days of ripening and storage as well as lower counts of thermophilic–mesophilic lactic acid bacteria. No differences were observed in the microbial counts between US and UG cheeses. Acetone, hexanal, 2 heptanone, ethanol and toluene were found in abundance in Urda cheeses. Both kashkaval of Pindos and Urda cheeses received high scores during the organoleptic evaluation. The obtained data may lead to the production of both cheeses with standard characteristics according to the traditional recipes and improve their recognition.

## 1. Introduction

Traditional cheeses are considered to be produced locally or regionally, usually seasonally, for many generations, and they have an important place in rural regional food culture. Furthermore, they reflect the country’s history, geography, climate and agriculture [1,2]. Nowadays, for the best valorisation and promotion of the local cheeses, it is important to produce them industrially while preserving their traditional character.

Numerous traditional cheeses are still made throughout Greece. Two of them are kashkaval of Pindos and Urda cheeses. Kashkaval of Pindos is a pasta filata milk cheese and Urda is a whey cheese. They are traditionally produced mainly at the high-altitude mountainous areas of Pindos as farmhouse cheeses intended for family consumption. Both cheeses are usually produced in summer during the seasonal migration (movement) of shepherds and their livestock from lower to higher altitude pastures.

In previous studies, the seasonal (usually during summer, at the end of the lactation period) manufacturing process of kashkaval of Pindos and Urda farmhouse cheeses made in small artisanal units using traditional technology and simple processing equipment in the mountains was described [3,4,5]. The present work was conducted to complement the previous studies.

The objective of the present study was (a) considering the nutritional properties of goat milk, to investigate the percentage of goat milk that can be added to sheep milk in order to produce kashkaval of Pindos cheese; (b) to utilize the whey from the manufacture of kashkaval of Pindos cheese in order to produce Urda whey cheese; and (c) to study the microbiological, textural, chemical and organoleptic characteristics of kashkaval of Pindos and Urda cheeses manufactured using sheep or a mixture of sheep–goat milk and the residual whey, respectively, at different sampling dates.

## 2. Materials and Methods

### 2.1. Preliminary Experiments

In the preliminary experiments, kashkaval of Pindos cheeses were manufactured, at the Dairy Research Department, Institute of Technology of Agricultural Products, using the procedure described elsewhere [3] with some modifications: coagulation took place at 35 °C and no dry salt was added during kneading of the curd. Pasteurized 100% sheep milk, 100% goat milk, a mixture of 90% sheep milk and 10% goat milk or of 70% sheep milk and 30% goat milk were used.

### 2.2. Cheese Manufacture and Sampling

**Figure 1:**
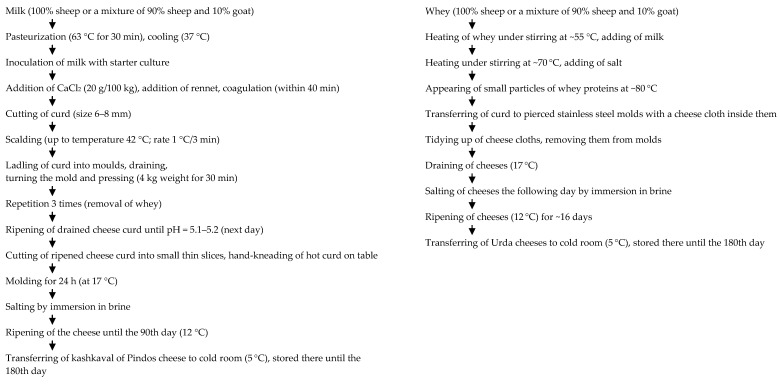
Manufacturing protocol of kashkaval of Pindos and Urda cheeses.

A flow diagram of the manufacturing process of kashkaval of Pindos and Urda cheeses is shown in Figure 1. Both cheeses were manufactured at the Dairy Research Department, taking into account their traditional cheese-making methods.

Two kashkaval of Pindos cheeses were produced using either 100% sheep milk (KS) or a mixture of 90% sheep and 10% goat milk (KG). After pasteurization, a freeze-dried, direct-to-vat set starter culture (RSF-736, Hansen) consisting of *Lactobacillus helveticus*, *Lactococcus lactis* subsp. *cremoris*, *Lactococcus lactis* subsp. *lactis* and *Streptococcus thermophilus* was used, following the manufacturer’s instructions. At a temperature of 35 °C, rennet (1:10,000 strength, NATUREN Extra NB, Hansen) was added according to the supplier’s instructions. Cheeses were salted by immersing them in brine (18°Be) for 4 days at 17 °C and then were transferred to a room with a lower temperature (12 °C) for maturation until they were 90 days old. At this point, kashkaval of Pindos cheeses were vacuum-packaged in polyethylene bags (Valko Favola 415/20, Italy) and transferred to cold rooms (5 °C) for storage until 180 days.

From the production of kashkaval of Pindos cheese, 100% sheep whey (US) or a mixture of 90% sheep whey and 10% goat whey (UG) was obtained. This whey was used for the manufacturing of Urda cheese. The whey was heated gradually under continuous stirring to 55 °C. At this temperature, a quantity (11 kg) of 100% sheep milk or a mixture of 90% sheep milk and 10% goat milk was added. Heating under stirring was continued; at 70 °C, salt (0.5% *w/v*) was added, and stirring was stopped at 80 °C. After draining, cheeses (approximately 0.6 kg) were salted by immersion in brine (18°Be) for one hour, at 17 °C. Then Urda cheeses were left to ripen at 12 °C until their moisture content reached 25–30% (approximately 16 days). They were then vacuum-packaged in polyethylene bags (Valko Favola 415/20, Italy) to avoid excessive moisture loss and mould growth, and they were stored at 5 °C until the 180th day.

Kashkaval of Pindos cheese samples were taken immediately after the kneading process (day 1), at the end of salting (6th day), and after 30, 60, 90 and 180 days of ripening and storage.

Urda cheese samples were taken for analyses at first day, the day cheeses entered the cold room (16th day), and 45, 90 and 180 days after cheese manufacture.

### 2.3. Gross Composition of Cheese

Cheese was examined for pH electrometrically (Micro pH 2002; Crison, Barcelona, Spain) and was analysed for: fat content according to the Gerber-Van Gulik method [6], salt content by the modified Volhard method [7] and protein content by multiplying the values of total nitrogen (TN) with the factor 6.38. Moisture was assessed by drying to constant weight at 102 ± 1 °C [8]. Ash content in cheese was measured following the method described by the Association of the Official Analytical Chemists standard 935.42 [9]. Yield was expressed in kg of cheese produced from 100 kg of milk.

### 2.4. Organoleptic Evaluation

The cheeses, cut in small cubes of ~2 cm side, were organoleptically assessed by five trained panel members who were permanent staff of the Dairy Research Department; all of them well experienced and familiar with pasta filata and whey cheeses. Kashkaval of Pindos cheese was assessed organoleptically at day 90 and 180, while Urda cheese was assessed at days 1, 16, 45, 90 and 180. Samples during evaluation had ambient temperature (18 ± 2 °C).

The panel was asked to evaluate the appearance (exterior, interior), texture (body) and flavour (odour and taste) and to notice any defects, according to the International Dairy Federation [10] guide for organoleptic evaluation of the cheese. All these attributes were graded on a 0–10 scale (0 = lowest quality, 10 = best quality). According to the importance given to each attribute, the scores for appearance were multiplied by a factor 1 (10 was the maximum score), for body texture by 4 (40 was the maximum score) and for flavour by 5 (50 was the maximum score). The total values were the sum of values of appearance, body texture and flavour. An excellent cheese received a total score of 100. Panellists used water to clean their mouth between samples and were unaware of the identity of the samples they tasted as samples were coded. Five replicate evaluations on each cheese sample were made.

### 2.5. Textural Analyses

The textural properties of mature (90, 180 days) cheese were analysed using a texture profile analyser (EZTest, EZ-X Series, Shimadzu Europa Gmbh, Germany) equipped with a 50 N load cell and a plunger with a diameter of 40 mm. The cheese sample had 20 mm × 20 mm × 20 mm dimensions and was compressed to 60% of its original height. From the compression curves, the following textural characteristics were calculated: (a) brittleness or fracturability (kg), b hardness (kg), (c) adhesiveness (kg × mm), (d) cohesiveness (ratio), (e) gumminess (kg). Data were collected using software TRAPEZIUM X (Shimadzu Autograph, Software, Shimadzu Europa Gmbh, Germany). Five replicate measurements of each cheese sample were made.

### 2.6. Proteolysis

Proteolysis was assessed by measuring different nitrogen fractions. Total nitrogen (TN) content was estimated by the Kjeldahl method [11]. Water-soluble nitrogen (WSN) and nitrogen soluble in 12% trichloroacetic acid (TCA) were determined in samples of extracts as described by Kuchroo and Fox [12]. The Sorval Omni-Mixer (Dupont Company, Newton, CT, USA) was used for homogenization and the supernatant was filtered through a No42 filter paper. Nitrogen soluble in 5% phosphotungstic acid (PTA) was also determined by the Kjelddahl method according to Stadhouders [13] with the exception that the extract was prepared as above mentioned.

### 2.7. Lipolysis

Lipolysis (total free fatty acid content) in cheeses was monitored at different ages using the method of Deeth and Fitz-Gerald [14]. Briefly, cheese (3 g) with water (5 mL) was transferred to test tubes and mixed with 10 mL of an extraction mixture (isopropanol: petroleum ether: 4 N sulfuric acid, 40:10:1). Petroleum ether (6 mL) was added and the tubes were shaken vigorously. The two layers were allowed to settle, and 5 mL of the upper layer was withdrawn and transferred to small flasks. Then, 6 drops of 1% methanolic phenolphthalein were added and the solution was titrated with the standard 0.02 N methanolic potassium hydroxide solution. From the titration, the Total Free Fatty Acid content was calculated.

### 2.8. Microbiological Analyses

On each sampling date, ten-gram portions of each cheese sample were added to 90 mL with sterilized Ringer solution ¼ strength and mixed with a stomacher (Bagmixer 400, Model VW, Interscience) for 120 s at room temperature. Subsequent dilutions were made in sterilised Ringer’s solution ¼ strength. Viable counts for staphylococci, lactic acid bacteria, lactic cocci, moulds and yeasts, coliforms and enterobacteria were performed in duplicate. A number of 1-mL or 0.1-mL samples of appropriate dilutions were poured or spread on total or selective agar plates for each species and according to instructions given by manufacturer. Unless otherwise stated, all media and supplements were purchased from Neogen Culture Media (Heywood, UK).

Coliform counts were enumerated on violet red bile agar after incubation at 30 °C for 24 h, and total Enterobacteriaceae were enumerated on violet red bile glucose agar after incubation at 37 °C for 24 h. Total mesophilic and thermophilic lactic acid bacteria (LAB) were enumerated on de Man, Rogosa, Sharpe (MRS) agar, incubated at 30 °C for 72 h under aerobic conditions and at 45 °C for 48 h. Mesophilic cocci and thermophilic cocci were enumerated on M17 agar, incubated at 30 °C for 72 h and 37 °C for 48 h, respectively. Total staphylococci were enumerated on Baird Parker agar base with egg yolk tellurite (BP), incubated at 37 °C for 48 h; yeasts and moulds were enumerated on rose bengal chloramphenicol agar, incubated at 25 °C for 5 days.

### 2.9. Volatile Compounds

The volatile compounds of cheese samples were studied by solid phase microextraction gas chromatography mass spectrometry (SPME-GC-MS) analysis. Homogenized 0.5 g cheese was placed in 10 mL glass vials fitted with a Teflon-lined septum sealed with an aluminium crimp seal through which an SPME syringe needle, equipped with a 2 cm fibre coated with 50–30 nm divinylbenzene-carboxen on dimethyl siloxane (DVB/CAR PDMS) bonded to a flexible fused silica core (Supelco, Bellefonte, PA, USA) was introduced. The fibre coating was exposed for 50 min at 60 °C to the cheese headspace under shaking in an automatic SPME autosampler (AOC 5000 Auto Injector, PAL, CTC Analytics, Zwingen, Switzerland). The absorbed volatile compounds were then analysed by GC-MS (GCMSQP2010, Shimadzu, Tokyo, Japan, capillary column Supelco CO Wax-10, 30 m length, 0.32 mm inside diameter and 0.50 μm film thickness). Helium was used as the carrier gas (linear velocity 0.8 mL min^−1^). The injector was operated at 260 °C and the oven was set to 45 °C for 5 min, then at a rate of 10 °C min^−1^ to 80 °C and finally at a rate of 5 °C min^−1^ to 240 °C for 10 min. The mass selective detector (QP2010, Shimadzu) operated in the electron impact mode with 70 eV electron energy and interface temperature of 270 °C. Semiquantification was performed by integrating the peak areas of total ion chromatograms (TIC) by the Shimadzu GCMS Solution software. The quantities of each compound were expressed as peak area ×10^3^ (in arbitrary units).

### 2.10. Statistical Analyses

The data were subjected to one-way analysis of variance to compare the values of each parameter of KS and KG or US and UG cheeses, at each sampling day. The software Statgraphics Plus for Windows v. 5.2 (Manugistics Inc., Rockville, MD, USA) was used and the means were separated by the Least Significant Difference (LSD) test at the 95% confidence level (*p* < 0.05). The same software was used to find a correlation between parameters.

## 3. Results and Discussion

### 3.1. Preliminary Experiments

The preliminary results have shown that kashkaval of Pindos cheese can be manufactured successfully using 100% sheep milk or by adding a small quantity of goat milk (10%). When mixture of 70% sheep milk–30% goat milk or 100% goat milk was used for kashkaval cheese production, the texture and stretching ability of the curd was of poor quality (tough curd that fractured during stretching).

### 3.2. Gross Composition of Cheeses

The physicochemical characteristics of kashkaval of Pindos cheese and of Urda cheese produced with the semi-industrial method in the pilot plant of the Dairy Department, are shown in Table 1 and Table 2, respectively. The mean composition of kashkaval of Pindos cheeses at 90 day of ripening and storage, i.e., age that can be sold in the market [15], ranged for moisture 41.25–41.83%, salt 2.00–2.05%, fat 27.58–28.50%, ash 4.24–4.38% and protein content 22.94–23.79% (Table 1). Generally, in the present study, there were no significant differences in pH values, in the content of moisture, salt, protein, ash, fat and in yield of KS and KG cheeses with the exception of at 180 days for ash and salt content (Table 1). Sheep (KS) cheeses (≥60 days) of this study fulfilled the requirements of European Economic Community (EEC) [16], i.e., fat-in-dry matter >45% and dry matter >58%. The results of this study were, in general, in accordance with the data found by others [17,18] for pasta filata cheeses. Similar pH and salt values to the present study but a higher moisture and protein content were reported for 90-day ovine kashar cheese [19]. At the same age, similar fat but a higher protein content was found in Caciocavallo cheese [20], whereas similar fat, protein and ash but lower moisture content of kashkaval cheese samples were observed [21]. The different types of milk, production methods and ripening conditions may explain the above differences. The cheese yield values of kashkaval of Pindos in this study were in accordance with the results of Pappa et al. [3]. At the end of storage (180 days) Pappa et al. [3] found lower values of pH and moisture content and higher values of fat content of pasteurized kashkaval cheese made from sheep milk in the mountains of Pindos than the values reported in this study; however those cheeses were not vacuum-packed, resulting in more moisture loss, subsequent decrease in pH values and an increase in fat content.

Regarding Urda chesses, generally, no statistical differences were found in the gross composition of US and UG cheeses at all sampling days (Table 2). However, when differences were observed, US cheeses showed higher fat and lower protein (*p* < 0.05) contents than UG cheeses. The gross composition of Urda cheeses in this study was generally within the range of that observed for whey cheeses [18,22]. Higher pH values were found for Anthotyro cheese [23], whereas lower pH values were assessed for Turkish traditional Mud whey cheese [24]. Furthermore, higher moisture content was found for Anthotyro and Manouri whey cheeses [23,25] than for those in the present study. At the first day of ripening, US cheeses of this study showed lower moisture, similar fat but higher protein and ash content than sheep-whey cheeses as reported by Kaminarides et al. [26]. The above differences could be attributed to the differences in whey and milk composition and different manufacturing conditions used. The cheese yield values were in accordance with the results of Kaminarides et al. [26]. Urda cheese of this study (Table 2) showed similar levels of moisture and salt but lower fat and higher protein content when compared to Urda cheese made traditionally in the mountains of Pindos [5]. According to the traditional manufacture protocol, the whey was obtained after the manufacturing of the hard cheese, using a procedure that included “beating” the coagulum to enrich the whey with fat [5]; therefore, traditional Urda cheese contained high levels of fat (43.5% for sheep cheese at 90 days of storage). The fat content (30.2% for sheep milk at the 90th day of ripening and storage) in this study was lower, as this was our aim due to the new trend of consuming less fat in human nutrition. Further studies are needed in order to investigate the percentage of milk that can be added to the whey in order to produce Urda cheese with similar fat content to the traditional cheese. Moreover, in the present study, US cheeses showed higher values of protein (30.18% at the 90th day) content than the traditional cheese (21.92% at the same age) [5] providing an increased nutritional value. It is known that whey protein content in essential amino acids is more than sufficient to cover the needs of a person [22].

### 3.3. Organoleptic Characteristics

Among the most important factors of the food quality are appearance, flavour and texture [27]. Therefore, cheese organoleptic characteristics are an important quality aspect which determines the consumer’s behaviour and the decision to purchase it. From the production method used in this study, both kashkaval of Pindos and Urda cheeses received very favourable grades for all the organoleptic characteristics, as shown in Table 3 and Table 4, respectively. In detail, mature (90–180 days) kashkaval of Pindos cheese received 9.11–9.40 scores for appearance, 36.20–37.50 for body texture and 44.64–47.24 for flavour for KS and KG cheeses, respectively. There were no quality defects described by the panellists in this study, such as red or dirty white rind, etc. as reported by Caric [28] for kashkaval cheese. Kashkaval of Pindos was general described as a cheese with a mild, mellow, and faintly sweet flavour.

Urda (Table 4) was very much appreciated as a fresh cheese (1–16 days) and received 8.46–9.33 scores for appearance, 34.82–37.23 for texture and 43.33–46.58 for flavour, and as a ripened cheese (45–180 days) it received 8.74–9.28, 34.11–37.11 and 42.57–45.33, for US and UG cheeses, respectively. Fresh Urda cheese had soft texture, off-white colour and pleasant flavour; while ripened cheese was yellow, hard, with a piquant and fairly salty taste and nice aroma. Fresh Urda cheese can be eaten as a table cheese, sometimes with sugar or honey, or it can be used for the preparation of certain foods and cheese pies; while ripened cheese was quite hard and suitable for grating [22].

Data regarding the organoleptic evaluation (Table 3) did not reveal statistical differences (*p* > 0.05) among mature KS and KG cheeses generally. However, at 180 days of ripening and storage, KG cheeses received significant higher flavour scores (*p* < 0.05) than KS cheeses. Regarding Urda cheeses, there were no statistical differences in the organoleptic characteristics, as shown in Table 4. However, at 90-day of storage, UG cheeses showed higher levels of flavour and total scores than US cheeses, but at 180 days, no differences were detected (*p* > 0.05). It can be seen that kashkaval of Pindos and Urda cheeses made with the addition of goat milk (KG or UG) were very much appreciated by the panellists.

### 3.4. Textural Analyses

Texture is widely recognized as an important quality factor as it affects usage properties (i.e., ease of cutting, grading, spreading), handling properties (i.e., shape retention), ease of curd fusion and rind and hole formation [29]. The textural characteristics of kashkaval of Pindos cheese are shown in Table 5, and to our knowledge, they are reported for the first time. At 90 days of ripening and storage, values for brittleness ranged between 0.011 and 0.037 kg, for hardness between 2.567 and 2.317 kg, for adhesiveness between −0.100 and −0.113 kg × m, for cohesiveness between 0.565 and 0.464 and for gumminess between 1.216 and 1.341 kg for KS and KG cheeses, respectively. There were no statistical differences (*p* > 0.05) in the textural characteristics of mature KS and KG cheeses, probably because no statistical differences were observed in their physicochemical content (moisture, fat and protein). Some differences (*p* < 0.05) observed in the salt content of KS and KG cheeses at 180 days of storage did not affect their textural properties (*p* > 0.05). Hardness of kashkaval of Pindos cheese was correlated with its protein content (correlation coefficient = 0.69, *p* < 0.05). A high correlation (*r* = 0.76) between hardness and the chemical composition of kashkaval cheese was reported by Kindstedt et al. [17].

However, no textural properties could be obtained for Urda cheese using load cell capacity 50 N in the texture profile analyser (as described in section Materials and Methods).

### 3.5. Proteolysis

During cheese ripening, many chemical changes occur in its principal constituents; proteins, lipids and residual lactose are degraded first to primary and then to secondary products. Proteolysis during ripening plays a vital role in the development of texture and contributes directly to flavour and off-flavour of a cheese. In order to understand the development of proteolysis in cheese, it is necessary to monitor the nitrogen fractions formed during ripening. Using different precipitation chemicals, it is possible to separate the nitrogen components in cheese into different soluble fractions [30]. Fractionation with water (WSN), with 12% trichloroacetic acid (TCA) and with 5% phosphotungstic acid (PTA) are commonly used as an index of the rate and extent of proteolysis. The 12% TCA soluble fraction contains small peptides (2–20 residues) and amino acids, while the 5% PTA soluble fraction contains low molecular weight peptides (<600 daltons) [30].

The nitrogenous fractions of kashkaval of Pindos cheese made using 100% sheep (KS) or a mixture of 90% sheep milk and 10% goat milk (KG), during ripening and storage, are shown in Table 6. The type of milk used for the manufacturing kashkaval of Pindos cheese did not affect the proteolysis level. In this study, at 90 days of ripening and storage, the proteolysis index (100 × WSN/TN) ranged from 14.59–18.52%, regardless of the type of milk used. A similar rate of the proteolysis index of kashkaval cheese was reported by others [3,18,28,31].

The PTA levels of KS and KG cheeses increased from 0.44–0.45% at day 1 of storage to 1.91–1.67% at day 180 of storage, respectively. Temizkan et al. [19] observed higher PTA values at 90-day old ovine kashar cheese.

The TCA values of KS cheeses increased from 0.99% at day 1 to 10.31% at day 180 and from 1.21% to 9.05% for KG cheeses, respectively. Similar TCA values were found at 90-day old kashkaval [32] and ovine kashar cheese [19].

Total nitrogen content and soluble nitrogen fractions of Urda cheeses are shown in Table 7. The WSN values in US and UG cheeses were 3.36–3.48% at day 1 and 3.58–3.27% at day 180, respectively. Additionally, the TCA and PTA values of US and UG cheeses were 2.02–1.83% and 0.99–1.08% at day 1, respectively. At day 180, these values were 1.89–1.79% and 0.86–0.84%, respectively, indicating that the levels of proteolysis were low during ripening and storage. Urda cheese in this study showed higher WSN content than Mud whey cheese, but lower TCA and PTA values than Lor and other whey cheeses [24,33,34].

The different technological parameters may explain the observed differences.

### 3.6. Lipolysis

As shown in Table 6, the total free fatty acid content of KS and KG cheeses increased from 0.66–0.67 meqv/g cheese at day 1 to 1.29–1.58 meqv/g cheese at day 180 in accordance with the results of Omar and El-Zayat [35] and Kindsted et al. [17].

The total free fatty acid content of US and UG is presented in Table 7 and it was doubled at the end of ripening and storage. In the literature, a moderate rate of lipolysis was reported for Manouri [25] and Mud whey cheese [24], whereas no statistical changes over time were found for Lor whey cheese [33].

In general, in this study, no differences were observed between KS and KG cheeses (Table 6) and between US and UG cheeses (Table 7).

### 3.7. Microbiological Analyses

Enumeration of microbial groups during ripening and storage of KS and KG cheese are presented in Table 8. Low counts of thermophilic and mesophilic lactic acid bacteria (LAB) were found at day 1 and during ripening and storage; this could be attributed to the ‘pasta filata’ process which involves immersion of the acidified curd slices in hot water, which might have inactivated them. These results are in accordance with the findings of Simov et al. [31] who found that hot-brining of kashkaval curd destroyed a very high proportion of the LAB starter. At all sampling dates, KG cheeses showed higher counts of mesophilic LAB than KS cheeses. Moreover, when differences were observed during ripening and storage, KG cheeses showed higher counts of thermophilic LAB as well as mesophilic and thermophilic cocci than KS cheeses (Table 8). Differences in the thermophilic and mesophilic microorganisms were also observed in Caciocavallo cheeses made from different types of milk [36]. Total staphylococcal colonies, Enterobacteriaceae, coliforms and yeasts-moulds of kashkaval of Pindos cheese were at low levels at all sampling dates (data not shown). The use of pasteurized milk and the heat treatment of the curd during texturizing possibly had a preservative effect on kashkaval of Pindos cheese, which is in agreement with the results of Pappa et al. [3] and Samelis et al. [4].

The microbiological data of US and UG cheeses during ripening and storage are shown in Table 9. Physical entrapment of bacteria in the curd and microbial growth during cheese drainage and salting were responsible for the microbial counts at day 1 [25,37]. Generally, no differences were observed in the microbial counts between US and UG cheeses during ripening and storage (Table 9). The high mean pH values, ranging from 6.39–5.82 at the different sampling dates (Table 2) of US and UG cheese were favourable for the growth of the different microbial groups. Generally, higher counts were found in other whey cheeses [5,23,24,25,33] compared to those in this study, reflecting possible differences in the microbial numbers of the whey and milk used for the cheese-making. The counts of total staphylococci, coliforms, Enterobacteriaceae and yeasts-moulds remained low (data not shown) possibly because of the combined effect of salt (the salt-in-moisture content was 13.97% ± 0.70% and 14.72% ± 3.02% for US and UG, respectively at 180 days; data not shown), low moisture content and low storage temperature in addition to the vacuum-packaging of the cheeses in polyethylene bags.

### 3.8. Volatile Compounds

In kashkaval of Pindos cheese, twenty-seven volatile compounds were identified; three hydrocarbons, five ketones, three aldehydes, eight alcohols, seven free fatty acids and one terpene (Table 10). Most of these compounds had already been reported by other researchers for the same cheese variety [19,38]. The KS cheese showed higher (*p* < 0.05) total volatile compounds than KG cheeses at 60 and 90 days of ripening and storage, whereas no differences were observed at 180 days (*p* > 0.05). Total hydrocarbons were only detected at day 60, total ketones remained stable (*p* > 0.05), total aldehydes decreased, total alcohols and total free fatty acids increased from day 60 to day 180 in KS cheese. In KG cheese, total hydrocarbons, total ketones and total alcohols increased (*p* < 0.05) from day 60 to day 180, while at the same time, total aldehydes decreased (*p* < 0.05) and total free fatty acids remained stable (*p* > 0.05). In the headspace of KS cheese, the most abundant compounds were 3methyl butanoic acid, 2methyl butanoic acid, phenylethyl alcohol and butanoic acid. In KG cheese, heptane, acetone and acetoin were found in abundance (Table 10). Butanoic acid has a strong, sweet odour which contributes very much to the cheese aroma and it was found in abundance in kashar [38] and kashkaval cheese [32]. In the literature, acetoin was found in abundance in kashar cheese [38]; while kashkaval cheese also showed high content of heptane [32]. Phenylethyl alcohol has a pleasant floral odour and may be produced from phenylalanine by microbial catabolism, whereas acetone generally originates either from milk or is produced from the thermal degradation of the β-ketoacids [39] and was found in abundance in the volatile fraction in kashkaval cheese [3]. The branched-chain fatty acids 2 and 3 methyl butanoic acids are probably produced from leucine and isoleucine, respectively; they provide sweet and fruity notes and were found in high concentrations in aged Italian cheeses [40].

Regarding Urda cheese, seventeen compounds, i.e., three hydrocarbons (tetradecane, hexadecane, toluene), three ketones (acetone, 2heptanone, 2nonanone), five alcohols (2propanol, ethanol, hexanol, hexanol 2ethyl, butanol 3methyl), five aldehydes (pentanal, hexanal, heptanal, octanal, nonanal) and one terpene (limonene) were identified (Table 11). Most of these compounds have been reported in other whey cheeses [26]. Furthermore, in general, these compounds were found in the headspace of the artisanal Urda cheese [5], however, more compounds were assessed in the traditional cheese produced in the mountains of Pindos than in this study. In the present work (Table 11), the US cheese showed lower total volatile compounds than UG cheeses at all sampling dates. From Table 11, it can be seen that ketones and aldehydes were the most abundant compounds in US cheese at the day 1 (fresh cheese) and ketones at day 180 (mature cheese). In UG cheeses, ketones, aldehydes and hydrocarbons were the major compounds in the fresh cheese (1-day old) and alcohols followed by ketones were the major compounds in the mature cheeses (180-day old). Acetone, hexanal and 2heptanone were found in abundance in US cheeses, while acetone, hexanal, ethanol and toluene were found in UG cheeses.

## 4. Conclusions

Kashkaval of Pindos and Urda are two popular traditional cheeses with a high cultural value and economic impact for the regions in which they are produced. The present work (a) investigated the development of an appropriate technology using partial substitution of sheep with goat milk, as there is a growing interest among consumers in goat cheeses. The results shown that only 10% goat milk could be added to sheep milk, as this percentage resulted in a product with proper characteristics and satisfactory acceptance by the consumers. Additionally, (b) in this study, whey from the production of kashkaval of Pindos cheese was not considered a by-product of cheese manufacturing and was not disposed of as waste effluent, but Urda whey cheese was produced with a manufacturing method based on the traditional one. Moreover, (c) the microbiological, textural, chemical and organoleptic results of the cheeses are useful in order to define their identity. The manufacturing method used resulted in no observed differences between KS and KG cheeses, as well as between US and UG cheeses regarding their physicochemical, textural characteristics, level of proteolysis and level of lipolysis. The fat content of Urda cheese in this study was lower than that of the artisanal cheese, which is in accordance with the new trend of consumers aiming to receive less fat in their nutrition. Moreover, both kashkaval of Pindos and Urda cheeses made using 100% sheep milk or a mixture of 90% sheep–10% goat milk and whey, respectively, received high scores during the organoleptic evaluation. The data of the present study can lead to the production of kashkaval of Pindos and Urda cheeses with standard characteristics, allowing their expansion in the domestic or international market by giving an added value to these local, traditional cheeses.

## Figures and Tables

**Table 1:** Changes of physicochemical characteristics of kashkaval of Pindos cheese made from 100% sheep (KS) or a mixture of 90% sheep milk–10% goat (KG) milk during ripening and storage.

Time (Days)	Cheese	pH	Moisture, %	Salt, %	Fat, %	Ash, %	Yield, %	Proteins, %
1	KS	5.16 ± 0.07 a	45.22 ± 0.27a	-	25.00 ± 0.76a	2.68 ± 0.14a	17.58 ± 0.68a	23.61 ± 1.25a
	KG	5.08 ± 0.04a	45.19 ± 0.06a	-	25.17 ± 1.01a	2.69 ± 0.05a	15.96 ± 0.27a	23.91 ± 1.21a
6	KS	5.33 ± 0.06a	43.97 ± 0.58a	0.28 ± 0.08a	27.43 ± 0.43a	2.89 ± 0.02a	17.21 ± 0.68a	23.23 ± 0.12a
	KG	5.34 ± 0.04a	44.57 ± 0.31a	0.32 ± 0.06a	26.67 ± 0.72a	2.91 ± 0.09a	15.61 ± 0.31a	23.02 ± 0.47a
30	KS	5.40 ± 0.03a	41.50 ± 0.16a	1.39 ± 0.04a	27.75 ± 0.25a	3.97 ± 0.07a	17.11 ± 0.66a	24.21 ± 0.28a
	KG	5.39 ± 0.08a	42.01 ± 0.45a	2.06 ± 0.08b	27.92 ± 0.33a	4.20 ± 0.09a	15.46 ± 0.31a	23.72 ± 0.40a
60	KS	5.55 ± 0.07a	41.39 ± 0.56a	2.23 ± 0.18a	27.67 ± 0.22a	4.37 ± 0.08a	16.11 ± 0.71a	23.58 ± 0.50a
	KG	5.38 ± 0.06a	41.39 ± 0.35a	2.11 ± 0.08a	27.50 ± 0.25a	4.33 ± 0.07a	15.63 ± 0.54a	23.69 ± 0.35a
90	KS	5.50 ± 0.00a	41.83 ± 0.37a	2.00 ± 0.13a	28.50 ± 0.29a	4.24 ± 0.04a	16.09 ± 0.70a	22.94 ± 0.60a
	KG	5.44 ± 0.04a	41.25 ± 0.40a	2.05 ± 0.05a	27.58 ± 0.30a	4.38 ± 0.10a	15.42 ± 0.34a	23.79 ± 0.46a
180	KS	5.47 ± 0.01a	41.85 ± 0.06a	2.28 ± 0.08a	27.00 ± 0.25a	4.65 ± 0.06a	15.69 ± 1.0a	24.36 ± 1.22a
	KG	5.41 ± 0.03a	41.23 ± 0.24a	1.99 ± 0.02b	27.42 ± 0.17a	4.39 ± 0.41b	15.42 ± 0.33a	23.38 ± 0.39a

Means of three cheese-making trials ± standard error; a, b: Means for each parameter in the same column and sampling day with different letters significantly differ (*p <* 0.05); -: not measured.

**Table 2:** Changes of physicochemical characteristics of Urda cheese made from 100% sheep (US) or a mixture of 90% sheep–10% goat (UG) whey during ripening and storage.

Time (Days)	Cheese	pH	Moisture, %	Salt, %	Fat, %	Ash, %	Yield, %	Proteins, %
1	US	6.39 ± 0.03a	60.1 ± 0.4a	1.5 ± 0.1a	16.3 ± 0.2a	3.36 ± 0.34a	7.9 ± 0.4a	16.8 ± 0.2a
	UG	6.33 ± 0.04a	60.4 ± 0.6a	1.7 ± 0.2a	15.2 ± 0.3b	3.31 ± 0.24a	7.8 ± 0.1a	17.7 ± 0.3a
16	US	6.07 ± 0.31a	28.2 ± 1.2a	3.4 ± 0.3a	30.3 ± 1.3a	6.17 ± 0.54a	4.3 ± 0.3a	30.1 ± 0.8a
	UG	5.75 ± 0.16a	28.6 ± 1.5a	3.9 ± 0.3a	26.9 ± 0.8b	6.73 ± 042a	4.4 ± 0.1a	32.1 ± 1.0a
45	US	5.98 ± 0.08a	27.7 ± 0.9a	3.4 ± 0.6a	30.1 ± 0.7a	5.99 ± 0.64a	4.5 ± 0.2a	29.9 ± 0.4a
	UG	6.01 ± 0.07a	28.3 ± 1.8a	3.8 ± 0.4a	27.3 ± 1.3a	6.57 ± 0.46a	4.3 ± 0.1a	32.0 ± 0.7a
90	US	5.86 ± 0.15a	26.7 ± 0.7a	3.7 ± 0.5a	30.2 ± 1.1a	6.36 ± 0.54a	4.5 ± 0.3a	30.2 ± 0.2a
	UG	5.98 ± 0.02a	27.9 ± 1.7a	3.9 ± 0.3a	25.8 ± 0.1b	6.70 ± 0.34a	4.1 ± 0.3a	33.3 ± 0.5b
180	US	5.96 ± 0.09a	27.2 ± 1.3a	4.1 ± 0.6a	29.6 ± 0.9a	6.31 ± 0.55a	4.5 ± 0.3a	30.5 ± 0.7a
	UG	5.82 ± 0.07a	28.1 ± 1.3a	3.9 ± 0.0a	26.5 ± 0.4b	6.46 ± 0.07a	4.4 ± 0.1a	32.9 ± 0.4b

Means of three cheese-making trials ± standard error; a, b: Means for each parameter in the same column and sampling day with different letters significantly differ (*p* < 0.05).

**Table 3:** Changes of organoleptic characteristics of mature kashkaval of Pindos cheese made from 100% sheep (KS) or a mixture of 90% sheep milk–10% goat (KG) milk during storage.

Time (Days)	Cheese	Appearance (10)	Body Texture (40)	Flavour (50)	Total (100)
90	KS	9.11 ± 0.03a	36.20 ± 0.29a	44.64 ± 0.43a	89.96 ± 0.65a
	KG	9.40 ± 0.14a	37.00 ± 0.50a	46.46 ± 0.55a	92.77 ± 1.19a
180	KS	9.33 ± 0.03a	37.5 ± 0.10a	46.28 ± 0.22a	93.10 ± 0.30a
	KG	9.37 ± 0.07a	37.49 ± 0.15a	47.24 ± 0.14b	94.33 ± 0.46a

Means of three cheese-making trials ± standard error. Mean values in brackets show the maximum scores. Total (100) is the sum of values of appearance (10), body texture (40) and flavour (50). a, b: means for each parameter in the same column and sampling day with different letters significantly differ (*p* < 0.05).

**Table 4:** Changes of organoleptic characteristics of Urda cheese made from 100% sheep (US) or a mixture of 90% sheep–10% goat (UG) whey during ripening and storage.

Time (Days)	Cheese	Appearance (10)	Body Texture (40)	Flavour (50)	Total (100)
1	US	9.12 ± 0.04a	35.84 ± 0.84a	44.58 ± 0a	89.55 ± 0.88a
	UG	9.33 ± 0.08a	37.23 ± 0.40a	46.58 ± 0.33b	92.97 ± 0.92a
16	US	8.46 ± 0.21a	34.93 ± 0.48a	43.82 ± 0.44a	87.21 ± 0.11a
	UG	8.85 ± 0.02a	34.82 ± 0.29a	43.33 ± 0.42a	86.73 ± 0.59a
45	US	9.01 ± 0.26a	35.11 ± 0.95a	43.11 ± 1.6a	87.23 ± 2.81a
	UG	8.74 ± 0.11a	34.11 ± 0.70a	42.57 ± 0.59a	85.42 ± 1.36a
90	US	8.78 ± 0.11a	34.65 ± 0.57a	42.61 ± 0.45a	86.05 ± 1.10a
	UG	9.25 ± 0.32a	36.5 ± 0a	45.32 ± 0.32b	91.07 ± 0.32b
180	US	9.28 ± 0.04a	37.11 ± 0.16a	43.24 ± 3.12a	93.44 ± 0.58a
	UG	9.16 ± 0.07a	36.63 ± 0.17a	45.33 ± 0.33a	91.13 ± 0.50a

Means of three cheese-making trials ± standard error. Mean values in brackets show the maximum scores. Total (100) is the sum of values of appearance (10), body texture (40) and flavour (50). a, b: means for each parameter in the same column and sampling day with different letters significantly differ (*p* < 0.05).

**Table 5:** Changes of textural characteristics of mature kashkaval of Pindos cheese made from 100% sheep (KS) or a mixture of 90% sheep milk–10% goat (KG) milk during storage.

Time (Days)	Cheese	Brittleness (kg)	Hardness (kg)	Adhesiveness (kg × mm)	Cohesiveness	Gumminess (kg)
90	KS	0.037 ± 0.027a	2.317 ± 0.103a	−0.100 ± 0.018a	0.565 ± 0.065a	1.341 ± 0.218a
	KG	0.011 ± 0.003a	2.567 ± 0.518a	−0.113 ± 0.051a	0.465 ± 0.05a	1.216 ± 0.362a
180	KS	0.065 ± 0.019a	2.636 ± 0.38a	−0.097 ± 0.008a	0.336 ± 0.02a	0.955 ± 0.099a
	KG	0.060 ± 0.016a	2.741 ± 0.53a	−0.130 ± 0.047a	0.367 ± 0.037a	1.083 ± 0.355a

Means of three cheese-making trials ± standard error. a: means for each parameter in the same column and sampling day with the same letter do not significantly differ (*p* > 0.05).

**Table 6:** Changes of total nitrogen, soluble nitrogenous fractions and total free fatty acid content of kashkaval of Pindos cheese made from 100% sheep milk (KS) or a mixture of 90% sheep milk and 10% goat (KG) milk during ripening and storage.

Time (Days)	Cheese	TN%	WSN, %TN	TCA, %TN	PTA, %TN	TFFA (μeqv g^−1^)
1	KS	3.7 ± 0.2a	2.5 ± 0.2a	1.0 ± 0.1a	0.5 ± 0.4a	0.7 ± 0.0a
	KG	3.8 ± 0.2a	2.7 ± 0.3a	1.2 ± 0.1a	0.4 ± 0.1a	0.7 ± 0.0a
6	KS	3.6 ± 0.0a	4.5 ± 0.9a	1. 5 ± 0.3a	0.6 ± 0.1a	1.0 ± 0.0a
	KG	3.6 ± 0.1a	7.2 ± 0.4a	2.8 ± 0.3a	1.0 ± 0.1a	0.9 ± 0.1a
30	KS	3.8 ± 0.0a	9.9 ± 0.8a	4.3 ± 0.7a	0.7 ± 0.1a	1.2 ± 0.1a
	KG	3.7 ± 0.1a	12.3 ± 1.2a	4.5 ± 0.2a	1.4 ± 0.3a	1.2 ± 0.0a
60	KS	3.7 ± 0.1a	12.6 ± 0.6a	4.6 ± 0.1a	0.7 ± 0.1a	1.1 ± 0.0a
	KG	3.7 ± 0.1a	15.8 ± 1.8a	5.2 ± 1.0a	1.5 ± 0.4a	1.1 ± 0.0a
90	KS	3.5 ± 0.0a	14.6 ± 1.4a	4.6 ± 0.3a	1.6 ± 0.1a	1.1 ± 0.1a
	KG	3.7 ± 0.1a	18.5 ± 2.1a	6.4 ± 0.9a	1.6 ± 0.3a	1.2 ± 0.0a
180	KS	3.8 ± 0.2a	21.2 ± 0.3a	10.3 ± 1.9a	1.9 ± 0.1a	1.3 ± 0.1a
	KG	3.7 ± 0.1a	20.6 ± 1.8a	9.1 ± 2.9a	1.7 ± 0.5a	1.6 ± 0.3a

Means of three cheese-making trials ± standard error. TΝ—total nitrogen; WSN—water-soluble nitrogen; TCA—nitrogen soluble in 12% trichloroacetic acid; PTA—nitrogen soluble in 5% phosphotungstic acid; TFFA—total free fatty acids. a: means for each parameter in the same column and sampling day with the same letter do not significantly differ (*p* > 0.05).

**Table 7:** Changes of total nitrogen, soluble nitrogenous fractions and total free fatty acid content of Urda cheese made from 100% sheep (US) or a mixture of 90% sheep–10% goat (UG) whey during ripening and storage.

Time (Days)	Cheese	TN%	WSN, %TN	TCA, %TN	PTA, %TN	TFFA (μeqv g^−1^)
1	US	2.6 ± 0.0 a	3.4 ± 0.1a	2.0 ± 0.2a	1.0 ± 0.1a	0.2 ± 0.0a
	UG	2.8 ± 0.1 a	3.5 ± 0.1a	1.8 ± 0.2a	1.0 ± 0.2a	0.2 ± 0.0a
16	US	4.7 ± 0.1a	3.5 ± 0.1a	1.9 ± 0.0a	1.1 ± 0.0a	0.4 ± 0.0a
	UG	5.0 ± 0.2a	3.4 ± 0.2a	1.7 ± 0.1a	1.0 ± 0.0a	0.3 ± 0.0b
45	US	4.7 ± 0.1a	3.5 ± 0.1 a	1.9 ± 0.1a	0.9 ± 0.1a	0.4 ± 0.1a
	UG	5.0 ± 0.1a	3.5 ± 0.2a	1.8 ± 0.1a	1.0 ± 0.1a	0.4 ± 0.0a
90	US	4.7 ± 0.1a	4.0 ± 0.4a	2.2 ± 0.4a	1.4 ± 0.3a	0.5 ± 0.0a
	UG	5.2 ± 0.2a	3.4 ± 0.1a	1.8 ± 0.0a	1.0 ± 0.0a	0.4 ± 0.0a
180	US	4.8 ± 0.1a	3.6 ± 0.1a	1.9 ± 0.1a	0.9 ± 0.0a	0.5 ± 0.0a
	UG	5.2 ± 0.1b	3.3 ± 0.1a	1.8 ± 0.1a	0.8 ± 0.0a	0.6 ± 0.0a

Means of three cheese-making trials ± standard error. TΝ—total nitrogen; WSN—water-soluble nitrogen; TCA—nitrogen soluble in 12% trichloroacetic acid; PTA—nitrogen soluble in 5% phosphotungstic acid; TFFA —total free fatty acids. a, b: means for each parameter in the same column and sampling day with different letters significantly differ (*p* < 0.05).

**Table 8:** Microbial changes (log cfu g^−1^) of kashkaval of Pindos cheese made from 100% sheep milk (KS) or a mixture of 90% sheep milk and 10% goat (KG) milk during ripening and storage.

Time (Days)	Cheese	Mesophilic Lactic Acid Bacteria	Thermophilic Lactic Acid Bacteria	Mesophilic Dairy Cocci	Thermophilic Dairy Cocci
1	KS	2.00 ± 0.00a	2.97 ± 0.34a	7.36 ± 0.24a	6.16 ± 1.08a
	KG	3.00 ± 0.09b	4.30 ± 0.17b	7.16 ± 0.16a	6.92 ± 0.23a
6	KS	2.32 ± 0.02a	2.25 ± 0.35a	4.16 ± 0.58a	4.66 ± 0.37a
	KG	3.54 ± 0.03b	4.31 ± 0.48b	5.78 ± 0.47a	6.15 ± 0.34b
30	KS	2.18 ± 0.00a	2.40 ± 0.00a	5.07 ± 0.39a	4.90 ± 0.32a
	KG	3.31 ± 0.01b	3.70 ± 0.52a	5.58 ± 0.06a	5.53 ± 0.30a
60	KS	3.36 ± 0.13a	2.87 ± 0.40a	4.14 ± 0.11a	4.86 ± 0.39a
	KG	5.99 ± 0.26b	3.61 ± 0.49a	5.58 ± 0.07b	5.71 ± 0.52a
90	KS	2.64 ± 0.09a	2.76 ± 0.38a	4.30 ± 0.39a	4.04 ± 0.48a
	KG	5.75 ± 0.85b	3.34 ± 0.35a	4.54 ± 0.57a	5.66 ± 0.46b
180	KS	2.60 ± 0.02a	2.55 ± 0.09a	4.59 ± 0.05a	4.05 ± 0.05a
	KG	5.54 ± 0.15b	3.31 ± 0.28a	5.52 ± 0.39a	4.60 ± 0.29a

Means of three cheese-making trials ± standard error. a, b: means for each parameter in the same column and sampling day with different letters significantly differ (*p* < 0.05).

**Table 9:** Microbial changes (log cfu g^−1^) of Urda cheese made from 100% sheep (US) or a mixture of 90% sheep–10% goat (UG) whey during ripening and storage.

Time (Days)	Cheese	Mesophilic Lactic Acid Bacteria	Thermophilic Lactic Acid Bacteria	Mesophilic Dairy Cocci	Thermophilic Dairy Cocci
1	US	3.41 ± 0.04a	4.34 ± 0.18a	3.49 ± 0.04a	4.05 ± 0.29a
	UG	3.60 ± 0.14a	3.46 ± 0.13b	3.36 ± 0.22a	3.70 ± 0.18a
16	US	4.04 ± 0.19a	3.62 ± 0.24a	4.53 ± 0.09a	5.26 ± 0.16a
	UG	4.58 ± 0.05b	4.08 ± 0.35a	5.18 ± 0.38a	5.32 ± 0.22a
45	US	4.36 ± 0.10a	4.70 ± 0.08a	4.13 ± 0.44a	4.74 ± 0.01a
	UG	4.63 ± 0.05a	4.90 ± 0.07a	4.83 ± 0.08a	4.64 ± 0.07a
90	US	5.00 ± 0.29a	4.84 ± 0.09a	6.01 ± 0.21a	6.26 ± 0.29a
	UG	5.37 ± 0.08a	5.71 ± 0.05b	6.26 ± 0.06a	6.14 ± 0.11a
180	US	4.00 ± 0.17a	4.02 ± 0.07a	5.58 ± 0.04a	5.67 ± 0.13a
	UG	3.85 ± 0.22a	3.58 ± 0.5a	5.89 ± 0.22a	5.93 ± 0.12a

Means of three cheese-making trials ± standard error. a, b: means for each parameter in the same column and sampling day with different letters significantly differ (*p* < 0.05).

**Table 10:** Volatile compounds (peak area ×10^3^) of kashkaval of Pindos cheese made from 100% sheep milk (KS) or a mixture of 90% sheep milk–10% goat (KG) milk during ripening and storage.

Volatile Compound	60 Day		90 Day		180 Day	
	KS	KG	KS	KG	KS	KG
HYDROCARBONS						
Heptane	2322.35 ± 358.38a	1830.3 ± 180.3a				27,380.9 ± 8851.45
Octane		894.8 ± 0		589 ± 167.04		548.8 ± 125
Toluene	226.6 ± 15.1a	372.35 ± 81.45a		381.5 ± 53.78		1577.8 ± 584.9
Total hydrocarbons	2548.95 ± 159.55a	3097.45 ± 57.07b		970.5 ± 194.08		29,507.5 ± 5412.36
KETONES						
Acetone	741.27 ± 88.88a	825.6 ± 154.9a	880.2 ± 46.5a	1332.4 ± 80.66b	1318.33 ± 326.32a	20,557 ± 7223.8b
2 Heptanone	1402.43 ± 36.29a	1295.7 ± 195.55a	2117.5 ± 189.5a	2652.87 ± 1200.01a	2745.8 ± 173.57a	1682.27 ± 269.33b
2 Pentanone	3664.07 ± 330.36a	2890.2 ± 613.47a	2439.73 ± 676.66a	2704.93 ± 121.89a	2061.8 ± 174.23a	2066.63 ± 409.14a
Acetoin	5314.5 ± 1095.67a	14,167.0 ± 1760b	4042.17 ± 326.84a	10,391.7 ± 1812.33b	3547.33 ± 809.73a	9458.43 ± 1615.07b
2 Nonanone	292.0 ± 7.83a	312.47 ± 41.24a	493.0 ± 50.1a	544.9 ± 199.55a	623.4 ± 25.23a	431.47 ± 44.23b
Total ketones	11,414.3 ± 1148.63a	19,491.0 ± 2573.83b	9972.6 ± 1020.45a	17,626.8 ± 617.60b	10,296.7 ± 811.76a	34,195.8 ± 2879.1b
ALDEHYDES						
Hexanal	299.35 ± 26.75a	323.4 ± 0.8a	191.3 ± 34.23a	413.7 ± 78.31a		214.2 ± 0
Nonanal	214.1 ± 32.34a	208.9 ± 56.26a	216.53 ± 16.93a	277.1 ± 49.69a	339.13 ± 29.81a	270.2 ± 32.0a
Heptanal	355.25 ± 151.65a	413.1 ± 56.22a	254.4 ± 0a	391.43 ± 40.35a	252.4 ± 0a	288.45 ± 31.85a
Total aldehydes	868.7 ± 75.23a	945.4 ± 47.09b	662.23 ± 26.69a	1082.23 ± 142.29b	591.53 ± 29.81a	772.85 ± 14.73b
ALCOHOLS						
Butanol, 3methyl	1948.67 ± 178.5a	1249.6 ± 703.4a	2298.67 ± 49.78a	2335.3 ± 409.4a	3017.77 ± 483.31a	2002.1 ± 152.2a
Pentanol	463.0 ± 61.52a	880.2 ± 231.1a	414.2 ± 104.3a	535.0 ± 60.1a	168.0 ± 41.8a	410.9 ± 41.5a
Hexanol		137.8 ± 11.6	1061.5 ± 0 a	89.4 ± 0a	241.4 ± 121.2	
2 Butanol		413.95 ± 157.25		270.8 ± 0		
Phenylethyl alcohol	6959.9 ± 815.2a	1836.25 ± 764.85b	8698.85 ± 891.35a	4887.1 ± 959.8a	13,769.8 ± 1319.87a	4927.3 ± 1047.4b
Ethanol	1114.9 ± 257.45a	2083.23 ± 443.01a	2874.43 ± 385.04a	1892.73 ± 139.87a	7545.9 ± 396.5a	2116.4 ± 327.33b
Hexanol, 2ethyl	266.0 ± 11		379.9 ± 0a	226.65 ± 53.05a		298.97 ± 20.72
Butanol			225.2 ± 0		255.1 ± 0	
Total alcohols	10,752.5 ± 94.93a	6601.03 ± 505.86b	15,952.8 ± 349.05a	10,237.0 ± 314.8b	24,998.0 ± 1744.11a	9755.67 ± 893.18b
FREE FATTY ACIDS						
Propanoic acid, 2methyl	3636.63 ± 766.22a	2314.9 ± 0a	3558.8 ± 1142.9a	1486.6 ± 560.7a	5052.8 ± 1708.5a	2061.3 ± 814.4a
Butanoic acid, 2 methyl	7317.6 ± 429.2a	1667.7 ± 3.3b	9961.35 ± 2839.85a	1880.55 ± 34.95a	24,456.6 ± 3531.42a	2256.15 ± 350.85b
Butanoic acid, 3methyl	6817.7 ± 2756.4a	4928.25 ± 32.55a	7821.7 ± 2.1a	4091.3 ± 972.22a	26,031.7 ± 2381.9a	3465.6 ± 691.8b
Hexanoic acid	3494.3 ± 1190.8a	2094.93 ± 342.26a	3479.07 ± 826.92a	4079.5 ± 2837.3a	5315.05 ± 1585.65a	4191.97 ± 1231.09a
Hexanoic acid, 2 ethyl	137.9 ± 0		1114.6 ± 0a	1198.65 ± 153.25a		802.3 ± 115.4
Butyric acid	7814.2 ± 0				13,580.2 ± 2021.0	
Octanoic acid					1061.7 ± 0	
Total free fatty acids	29,218.3 ± 945.86a	11,005.8 ± 326.46b	25,935.5 ± 2413.69 a	12,736.6 ± 1527.0b	75,498.05 ± 2487.24a	12,777.3 ± 885.39b
TERPENES						
d-Limonene	203.7 ± 0		299.5 ± 0			
Total Volatile Compounds	55,006.4 ± 955.3a	41,140.6 ± 2494.07b	52,822.6 ± 2543.13a	42,653.1 ± 1202.46b	111,384.3 ± 4432.8a	87,009.1 ± 9003.3a

Means of three cheese-making trials ± standard error. a, b: means for each parameter in the same row and sampling day with different letters significantly differ (*p* < 0.05).

**Table 11:** Volatile compounds (peak area ×10^3^) of Urda cheese made from 100% sheep (US) or a mixture of 90% sheep–10% goat (UG) whey during ripening and storage.

Volatile Compound	1 Day		45 Day		180 Day	
	US	UG	US	UG	US	UG
HYDROCARBONS						
Tetradecane		2936.35 ± 398.29		1306.4 ± 237.75		1986.8 ± 0
Hexadecane	1111.4 ± 0					
Toluene	1657.25 ± 250.37a	11,334.7 ± 1810.86β	437.7 ± 44.46a	17,262.7 ± 0 b	291.25 ± 30.51	
Total hydrocarbons	2768.65 ± 250.37a	14,271.05 ± 2209.14b	437.7 ± 44.46a	18,569.1 ± 237.75b	291.25 ± 30.51a	1986.8 ± 0 b
KETONES						
Acetone	10,080.6 ± 484.69a	11,327.8 ± 133.25a	2045.45 ± 607.92a	16,956 ± 2331.25b	1745.13 ± 293.35a	1682.4 ± 0a
2 Heptanone	1960.9 ± 746.37a	2266.65 ± 201.41a	2245.17 ± 608.05a	3037.5 ± 225.22a	4748.53 ± 732.12a	3752.7 ± 0a
2 Nonanone	479.1 ± 30.83aA	661.2 ± 0 b	896.83 ± 155.91a	1659.95 ± 165.96b	1616.87 ± 284.73a	1393.1 ± 0a
Total ketones	12,520.6 ± 355.19a	14,255.65 ± 68.16b	5187.45 ± 251.68a	21,653.45 ± 2722.44b	8110.53 ± 1240.38a	6828.2 ± 0 a
ALDEHYDES						
Hexanal	8592.7 ± 1074.57a	6745.75 ± 1237.81a	9347.9 ± 1214.4a	2419.8 ± 409.46b	643.57 ± 109.37a	711.25 ± 31.09a
Nonanal	430.0 ± 50.86a	1375.45 ± 95.87b	391.8 ± 28.17a	994.6 ± 0b	250.43 ± 21.53a	1702.8 ± 0 b
Heptanal	1810.35 ± 97.20a	3299.45 ± 314.11b	870.5 ± 104.10a	1344.85 ± 252.56a	507.75 ± 89.81a	777.15 ± 60.42a
Pentanal	1288.0 ± 31.87a	2814.65 ± 264.34b	984.8 ± 262.52a	2499.8 ± 726.65a	973.75 ± 53.61a	1807.45 ± 102.97b
Octanal	541.4 ± 49.71		639.4 ± 0			
Total aldehydes	12,662.45 ± 1041.59a	14,235.3 ± 1912.13a	12,234.4 ± 1085.5a	7259.05 ± 883.55b	2375.5 ± 95.47a	4998.65 ± 132.3b
ALCOHOLS						
Butanol, 3methyl	2075.1 ± 120.20		940.65 ± 110.53		374.97 ± 107.50	
Hexanol	243.35 ± 43.04a	291.6 ± 17.26a	500.15 ± 215.79a	414.5 ± 80.42a	129.4 ± 0a	190.55 ± 0.95b
Ethanol	1678.2 ± 546.48a	5350.5 ± 0b	2375.0 ± 639.36a	10,489.4 ± 0b		8459.0 ± 0
Hexanol, 2ethyl	248.0 ± 38.22		251.25 ± 41.42		242.3 ± 22.07	
2Propanol	2510.6 ± 455.59		2205.75 ± 529.81			
Total alcohols	6755.25 ± 876.66a	5642.1 ± 17.26a	6272.8 ± 928.32a	10,903.9 ± 80.42b	746.67 ± 129.19a	8649.55 ± 0.95b
TERPENES						
d-Limonene	436.2 ± 142.05a	1061.05 ± 334.37a	427.0 ± 67.37a	776.55 ± 75.78b		
Total Volatile Compounds	35,143.15 ± 1377.34a	49,465.15 ± 545.97b	24,559.35 ± 1855.49a	59,162.05 ± 3999.94b	11,523.95 ± 1418.93a	22,463.2 ± 133.25b

Means of three cheese-making trials ± standard error. a, b: Means for each parameter in the same row and sampling day with different letters significantly differ (*p* < 0.05).

## References

[1] Weichselbaum E., Benelam B., Costa H.S. (2009). Synthesis Report No. 6: Traditional Foods in Europe.

[2] Trichopoulou A., Soukara S., Vasilopoulou E. (2007). Traditional foods: A science and society perspective. Trends Food Sci. Technol..

[3] Pappa E.C., Kondyli E., Samelis J. (2019). Microbiological and biochemical characteristics of Kashkaval cheese produced using pasteurized or raw milk. Int. Dairy J..

[4] Samelis J., Kakouri A., Kondyli E., Pappa E.C. (2019). Effects of curd heating with or without previous milk pasteurization on the microbiological quality and safety of craft-made ‘pasta filata’ Kashkaval cheese curds. Int. J. Dairy Technol..

[5] Pappa E.C., Samelis J., Kondyli E., Pappas A.C. (2016). Characterization of Urda whey cheese: Evolution of main biochemical and microbiological parameters during ripening and vacuum packaged cold storage. Int. Dairy J..

[6] Schneider C., Les Hoires C.J., Wyss S.A. (1954). Traite Pratique Des Assais Du Lait Et Du Controle Des Produits Laitieres.

[7] Kosikowski F.V. (1982). Cheese and Fermented Milk Foods.

[8] IDF (1982). Cheese and Processed Cheese, Determination of the Total Solids Content (Reference Method).

[9] AOAC (1995). Ash of cheese 935.42 method. Official Methods of Analysis EI.

[10] IDF (1987). Sensory Evaluation of Dairy Products.

[11] IDF, Milk (1993). Determination of Nitrogen Content.

[12] Kuchroo C.N., Fox P.F. (1982). Soluble nitrogen in Cheddar cheese. Comparison of the extraction procedures. Milchwissenschaft.

[13] Stadhouders J. (1960). The hydrolysis of proteins during the ripening of Dutch cheese. The enzymes and the bacteria involved. Neth. Milks Dairy J..

[14] Deeth H.C., Fitz-Gerald P. (1976). Lipolysis in dairy products: A review. Aust. J. Dairy Technol..

[15] Greek Codex Alimentarius (2009). Official Journal of the Hellenic Republic.

[16] EEC (1990). No 1225/90 as regards the description of Kashkaval cheese. Off J. Eur. Commun..

[17] Kindstedt P., Caric M., Milanovic S., Fox P.F., McSweeney P.L.H., Cogan T.M., Guinee T.P. (2004). Pasta-filata cheeses. Major Cheese Groups: Cheese: Chemistry, Physics and Microbiology.

[18] Alichanidis E., Polychroniadou A. (2008). Characteristics of major traditional regional cheese varieties of East-Mediterranean countries: A review. Dairy Sci. Technol..

[19] Temizkan R., Yasar K., Hayaloglou A.A. (2014). Changes during ripening in chemical composition, proteolysis, volatile composition and texture in Kahar cheese made using raw bovine, ovine or caprine milk. Int. J. Food Sci. Technol..

[20] Perna A., Intagkietta I., Simonetti A., Gambacorta E. (2015). Effect of genetic type on antioxidant activity of Caciocavallo cheese during ripening. J. Dairy Sci..

[21] Talevski G., Srbinovska S., Santa D., Mateva N. (2017). Influence of packing materials on Kashkaval quality. Mljekarstro.

[22] Kandarakis J.G. (1986). Traditional whey cheeses. Bull. IDF.

[23] Kalogridou-Vassiliadou D., Tzanetakis N., Litopoulou-Tzanetaki E. (1994). Microbiological and physicochemical characteristics of ‘Anthotyro’, a Greek traditional whey cheese. Food Microbiol..

[24] Kavas N., Kavas G. (2011). Some properties of traditional whey cheese (Mud cheese) produced in Turkey. J. Food Sci. Eng..

[25] Lioliou K., Litopoulou-Tzanetaki E., Tzanetakis N., Robinson R.K. (2001). Changes in microflora of manouri, a traditional Greek whey cheese, during storage. Int. J. Dairy Technol..

[26] Kaminarides S., Nestoratos K., Massouras T. (2013). Effect of added milk and cream on the physicochemical, rheological and volatile compounds of Greek whey cheeses. Small Rumin. Res..

[27] Rosenberg M., Wang Z., Chuang S.L., Shoemaker C.F. (1995). Viscoelastic property changes in Cheddar cheese during ripening. J. Food Sci..

[28] Caric M., Fox P.F. (1993). Ripened cheese varieties native to the Balkan countries. Cheese: Chemistry, Physics and Microbiology.

[29] Luyten H., Van Vliet T., Walstra P. (1991). Characterization of the consistency of Gouda cheese: Rheological properties. Neth. Milk Dairy J..

[30] Christensen T.M.I.E., Betch A.-M., Werner H. (1991). Chemical methods for evaluating proteolysis in cheese maturation. Bull. IDF.

[31] Simov Z.I., Simova E.D., Beshkova D.M. (2006). Impact of two starter cultures on proteolysis in Kashkaval cheese. World J. Microbiol. Biotechnol..

[32] Suleijmani E., Hayaloglu A.A. (2016). Influence of curd heating on proteolysis and volatiles of Kashkaval cheese. Food Chem..

[33] Kavaz A., Arslaner A., Bakirci I. (2012). Comparison of quality characteristics of Çökelek and Lor cheeses. Afr. J. Biotecnol..

[34] Dimitrellou D., Kandylis P., Mallouchos A., Komaitis M., Koutinas A., Kourkoutas Y. (2010). Effect of freeze-dried kefir culture on proteolysis in feta-type and whey cheeses. Food Chem..

[35] Omar M.M., El-Zayat A.I. (1986). Ripening changes of Kashkaval cheese made from cow’s milk. Food Chem..

[36] Niro S., Fratianni A., Tremonte P., Sorrentino E., Tipaldi L., Panfili G., Coppola R. (2014). Innovative Caciocavallo cheeses made from a mixture of cow milk with ewe or goat milk. J. Dairy Sci..

[37] Tatini S.R., Jezeski J.J., Morris H.A., Olson J.C., Gasman E.P. (1971). Production of enterotoxin A in Cheddar and Colby cheeses. J. Dairy Sci..

[38] Hayaloglou A.A. (2009). Volatile composition and proteolysis in traditionally produced mature Kashar cheee. Int. J. Food Sci. Technol..

[39] Urbah G. (1997). The flavor of milk and dairy products. II. Cheese: Contribution of volatile compounds. Int. J. Dairy Technol..

[40] HA J.K., Lindsay R.C. (1991). Volatile branched-chain fatty acids and phenolic compounds in aged Italian cheese flavors. J. Food Sci..

